# Impact of Fatty Pancreas on Postoperative Pancreatic Fistulae: A Meta-Analysis

**DOI:** 10.3389/fonc.2021.622282

**Published:** 2021-12-01

**Authors:** Lu Zhou, Wei-ming Xiao, Cheng-peng Li, Yi-wen Gao, Wei-juan Gong, Guo-tao Lu

**Affiliations:** ^1^ Pancreatic Center, Department of Gastroenterology, The Affiliated Hospital of Yangzhou University, Yangzhou University, Yangzhou, China; ^2^ Department of Gastroenterology, Jingmen No. 2 People’s Hospital, Hubei, China; ^3^ Institute of digestive diseases, The Affiliated Hospital of Yangzhou University, Yangzhou University, Yangzhou, China; ^4^ Key Laboratory of Carcinogenesis and Translational Research (Ministry of Education/Beijing), Department of Hepato-Pancreato-Biliary Surgery, Peking University Cancer Hospital & Institute, Beijing, China; ^5^ School of Nursing, Yangzhou University, Yangzhou, China

**Keywords:** pancrea, BMI - Body Mass Index, fatty pancreas, pancreatic cancer, pancreatic fistula (PF)

## Abstract

**Background:**

Soft pancreas is widely recognized as an important risk factor for the development of postoperative pancreatic fistula (POPF). Although fatty pancreas (FP) has not been formally defined as a cause of pancreatic fistula, existing research has shown that it can increase the incidence of POPF by increasing pancreatic tenderness; therefore, it may be a potential risk factor. This study aimed to discern whether FP was associated with POPF.

**Method:**

Two reviewers independently performed literature searches from five electronic databases. According to the established inclusion criteria, we extracted necessary data from the studies that met the criteria for further analysis. We pooled the odds ratios (ORs) from individual studies using a random-effects model to investigate the associations between POPF and the prognosis of FP.

**Result:**

A total of 11 studies involving 2484 individuals were included. The pooled prevalence of POPF was 18% (95% CI: 12-24%). Body mass index (BMI) was associated with a significantly increased risk of POPF (OR=3.55; 95% CI: 1.83, 6.86; P=0.0002; I²=0). FP was obviously associated with the occurrence of POPF (OR=3.75; 95% CI: 1.64, 8.58; P=0.002; I²=78).

**Conclusion:**

FP is closely associated with the development of POPF, and the early identification of these high-risk patients can help to reduce the incidence of POPF.

**Systematic Review Registration:**

The Registration URL link is (https://www.crd.york.ac.uk/PROSPERO/). The ID is “CRD42021265141”.

## Introduction

Along with rapid urbanization, a steady improvement in people’s living standards, and increasing environmental pollution, the incidence of cancer is increasing year over year. As a highly destructive malignant disease, pancreatic cancer ranks 4th among cancer-related causes of death. Even with advanced technology, which is rapidly developing modern society, the 5-year survival rate is still as low as 8.2% (1, 2). Many surgical methods are used to treat pancreatic cancer, namely, pancreaticoduodenectomy, distal pancreaticotomy, middle pancreaticoduodenectomy, and pylorus-preserving pancreaticoduodenectomy, the choice of which is based on the location of the tumor. Pancreaticoduodenectomy(PD) is the classic surgical approach for pancreatic cancer (3).

Postoperative pancreatic fistula(POPF) is defined as an abnormal connection between epithelium of the pancreatic duct and epithelium containing enzyme-rich fluid derived from pancreatic tissue (4). POPF is a major determinant of the incidence of serious postoperative complications and mortality after surgery of the pancreas and plays an important role in operation-related mortality, morbidity, and the average length of hospitalization. Statistically, the incidence rate ranges from 3% to 45% (5). Risk factors for POPF include a soft pancreas, small pancreatic ducts, and reduced regional blood supply (6).

With the prevalence of obesity, a new pathological pattern has gradually become apparent—the term “fatty pancreas” was coined to describe fatty deposits in pancreatic cells (7), with obesity being the main cause of fatty pancreas (FP) (8). It has been shown that FP may exacerbate the condition of acute pancreatitis and lead to pancreatic dysfunction related to insulin resistance and T2DM, and it has even been associated with the development of pancreatic cancer (9). Although FP has not yet been classified as the cause of POPF, researchers have found that patients with POPF have a higher fat score, and described that FP may be a potential risk factor for POPF more than a decade ago (10).

To date, no meta-analyses have been conducted to study the effect of FP on POPF, so we performed a meta-analysis to explore this issue.

## Methods

### Search Strategy

Up to July 2020, a comprehensive literature search of electronic databases was performed, including PubMed, Web of Science, Embase, Medline, and Scopus. The search keywords were (“pancreatic diseases” OR “pancreas” OR “pancreas”) AND (“fatty” OR “steatosis” OR “lipomatosis”) AND (“fistula” OR “leakage”). The list of references in the study was also manually searched to determine their potential study value.

### Selection Criteria

#### (i) Inclusion Criteria

(1) The subjects were patients with POPF.
(2) FP needed to be diagnosed at the same time.

#### (ii) Exclusion Criteria

(1) Studies that were not relevant to the subject matter were excluded.
(2) Non-human research was excluded.
(3) Studies with incomplete data or data that could not be combined were excluded.
(4) Case reports, meeting abstracts, reviews, narrative summaries, and meta-analyses were excluded.
(5) Studies that did not conform to the ISGPF classification criteria were excluded.

### Data Extraction

A data extraction table was created, and data were input from each study, including author, year, country, method of FP determination, the total number of individuals, mean age, proportion of males, mean BMI, and Newcastle-Ottawa Scale (NOS) score. The NOS score was used to evaluate the quality of the included studies. Two authors independently assessed the quality of the studies and compared the data to avoid subjective bias. A third author made the final decision on differences. According to the ISGPS definition, only grades B and C clinically relevant-postoperative pancreatic fistulas (CR-POPF) were included in the analysis (5).

### Quality Assessment

We used NOS scores to evaluate the quality of the studies. The range of NOS is 0-9 points. In this study, we defined a score ≥ 6 as indicative of a high-quality study. Two authors independently assessed the quality of the studies.

### Statistical Analysis

All statistical analyses were performed by using Stata SE14.0 and RevMan5.3 software. The I² statistics were utilized to determine the heterogeneity among all the studies. In the analysis, I² < 50% indicated low heterogeneity, and the results were analysed using a fixed-effects model. I² > 50% indicated substantial heterogeneity, and the results were analysed using a random-effects model. Publication bias was assessed using Egger’s and Begg’s tests. OR was used to express the estimates in this study. A p-value <0.05 was considered statistically significant in all analyses.

## Results

### Study Characteristics

**Figure 1:**
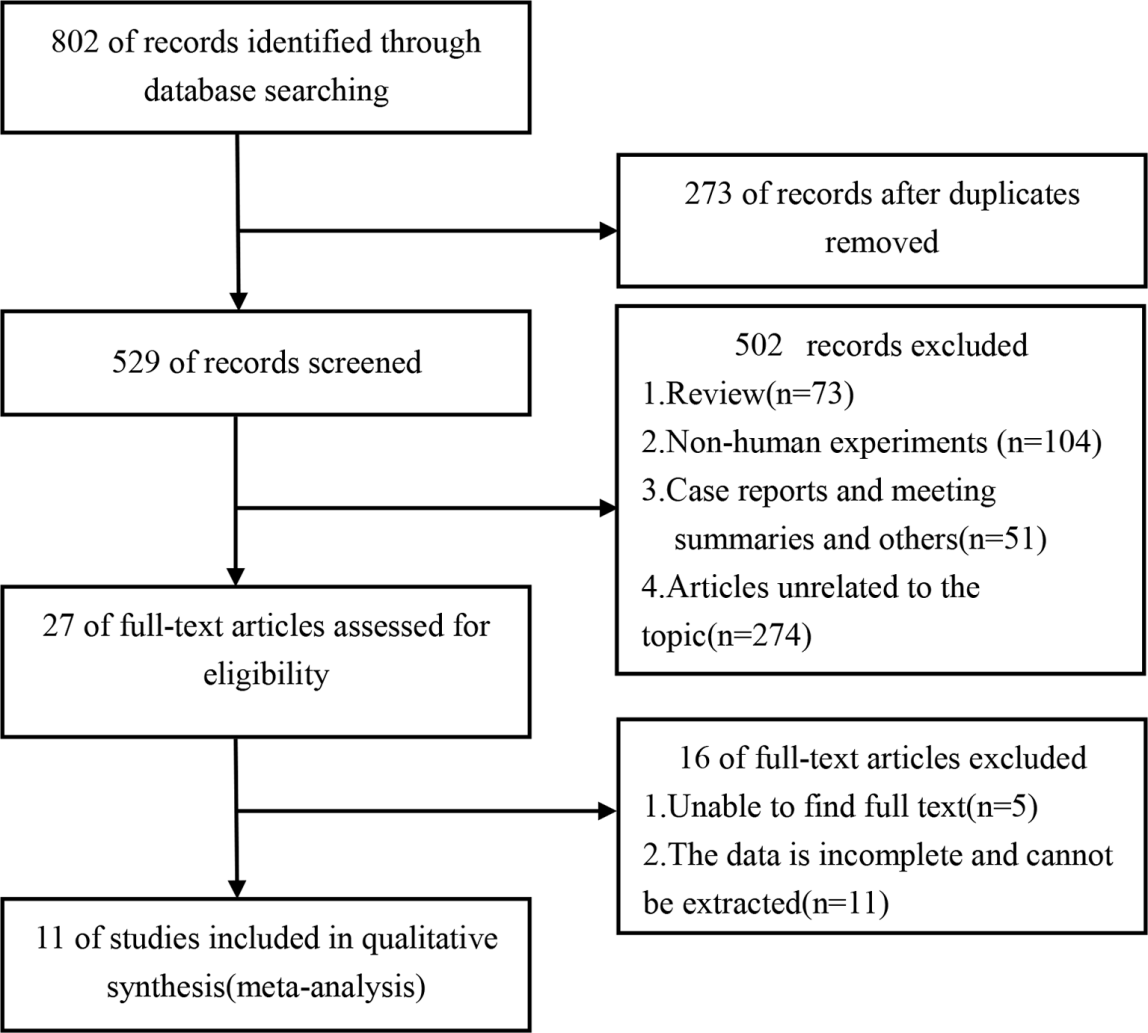
Flowchart of the study selection process.

The total number of articles retrieved from these five databases was 802. After removing duplicate entries (n=273), 529 articles were screened based on title and abstract. The full text of the 27 articles was assessed to apply the eligibility criteria. Eleven studies (encompassing 2484 individuals) were determined to be eligible for the meta-analysis (11–21) (**Figure 1**). The general clinical characteristics of the included studies are summarized in **Table 1**. All studies were retrospective and were published between 2009 and 2020. Five of the included studies focused on Asian patients, while the rest focused mainly on patients from Europe or North America. Eleven studies (including 2484) used the pathological method to determine FP (11–21). There were eight articles in which the number of individuals with FP was extractable (11–15, 17, 20, 21), and 5 (11, 14, 15, 17, 21) had the same diagnostic criteria for FP. The criteria were as follows: the total score of pancreatic fatty infiltration was obtained by the addition of both perilobular and intralobular scores. The score of fat infiltration around the perilobular and intralobular regions was as follows: 0: no fat infiltration; 1: partial adipocyte infiltration; and 2: massive adipocyte infiltration. A total score of 0-2 was regarded as fat-free, and a total score of 3 to 4 was regarded as fat infiltration (11, 14, 15, 17, 21).

**Table 1:** Characteristics of included studies.

Study	Year	Country	Method of FP determination	Total number of individual	Mean age, years	Men, n%	Mean BMI, kg/m^2^	NOS
Tanaka et al. (11)	2020	Japan	pathology	150	66	97,65%	22	6
Harrell et al. (12)	2020	America	pathology	301	63.2	150,49.8%	26.5	7
Xingjun et al. (13)	2019	China	pathology	609	NP	383,62.9%	NP	7
Patel and Yagnik (14)	2019	India	pathology	46	NP	30,65%	NP	6
Halle-Smith et al. (15)	2017	UK	pathology	107	67.5	59,55%	25.5	7
Yoon et al. (16)	2016	Korea	pathology	165	62.2	73,44%	NP	7
Tranchart et al. (17)	2012	France	pathology	103	58	58,56.3%	24	6
Belyaev et al. (18)	2011	Germany	pathology	696	62	383,55%	NP	6
Lee et al. (19)	2010	Korea	pathology	96	63.6	NP	NP	7
Rosso et al. (20)	2009	France	pathology	111	65	65,58.6%	NP	6
Gaujoux et al. (21)	2010	France	pathology	100	58	62,62%	24	7

BMI, body mass index; NOS, Newcastle-Ottawa Scale; NP,not reported.

### Quality Assessment of Included Studies

The quality of the included studies was evaluated using the NOS tool. Studies with NOS scores of 6 or higher were considered high-quality studies, while studies with NOS scores of 5 or less were considered low-quality studies. Based on the results of the quality assessment, the scores of the included studies ranged from 6 to 7 (**Table 1**), indicating that all included studies were of acceptable quality.

### Publication Bias

Publication bias was assessed by Egger’s and Begg’s tests. In articles reporting the prevalence of POPF (11–21), the P-values of each test were 0.436 and 0.324, respectively, indicating no evidence of publication bias in the included studies. Similarly, no bias was found in the study reporting the incidence of FP in people with POPF disease (11–15, 17, 20, 21), with the P-values of each test being 0.386 and 0.409, respectively.

### Prevalence of POPF

**Figure 2:**
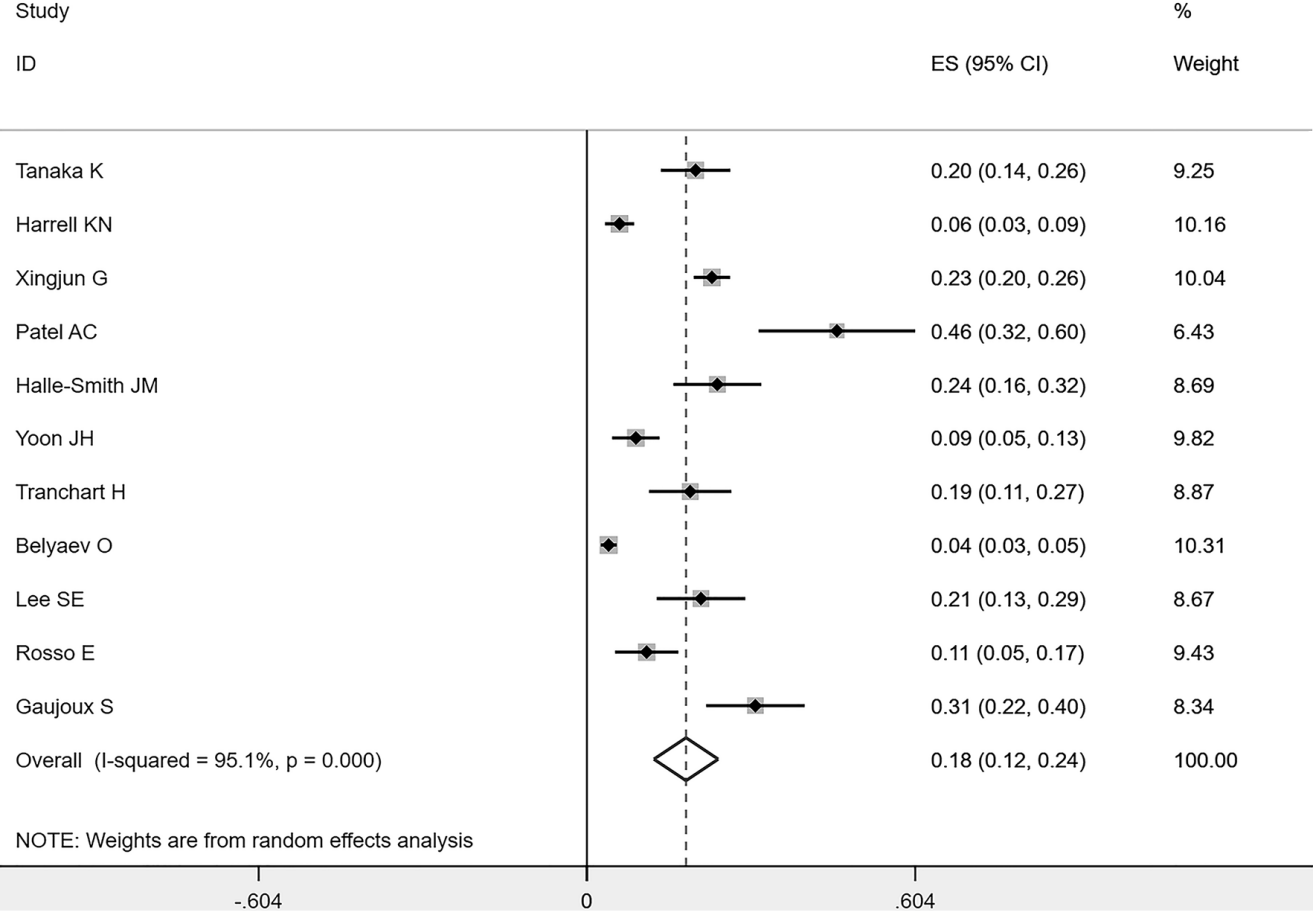
Forest plots of POPF’s prevalence rate.

After screening all the studies, a total of 11 studies (including 2484 individuals) explored POPF in patients who underwent pancreaticoduodenectomy (11–21). Therefore, the included studies could be applied to investigate prevalence in the meta-analysis. The pooled prevalence of POPF was 18%, and the 95% confidence interval was 12-24% (**Figure 2**).

### Association Between BMI and POPF

**Figure 3:**
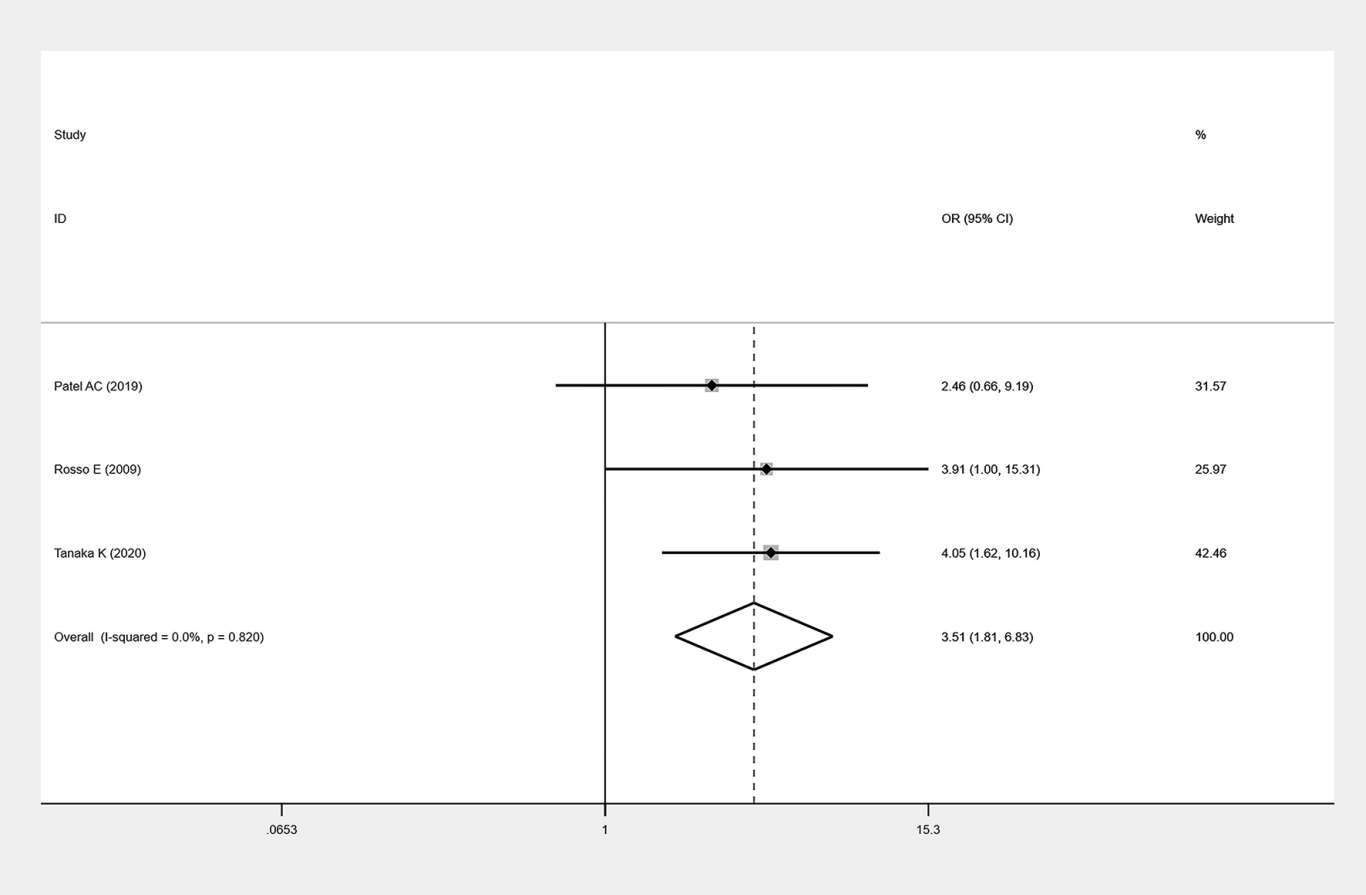
Forest plots of BMI on POFP.

A total of three studies assessed the effect of BMI on the incidence of POPF (11, 14, 20) and 307 individuals were included in the analysis. Among the 91 patients with POPF, 28 patients had a BMI >25 kg/m². Among the remaining 216 non-POPF patients, 63 patients had a BMI >25 kg/m². These results suggest that high BMI is associated with a significant increase in the occurrence of POPF (OR=3.51; 95%CI: 1.81,6.83; P=0.0002; I²=0) (**Figure 3**).

### Association Between FP and POPF

**Figure 4:**
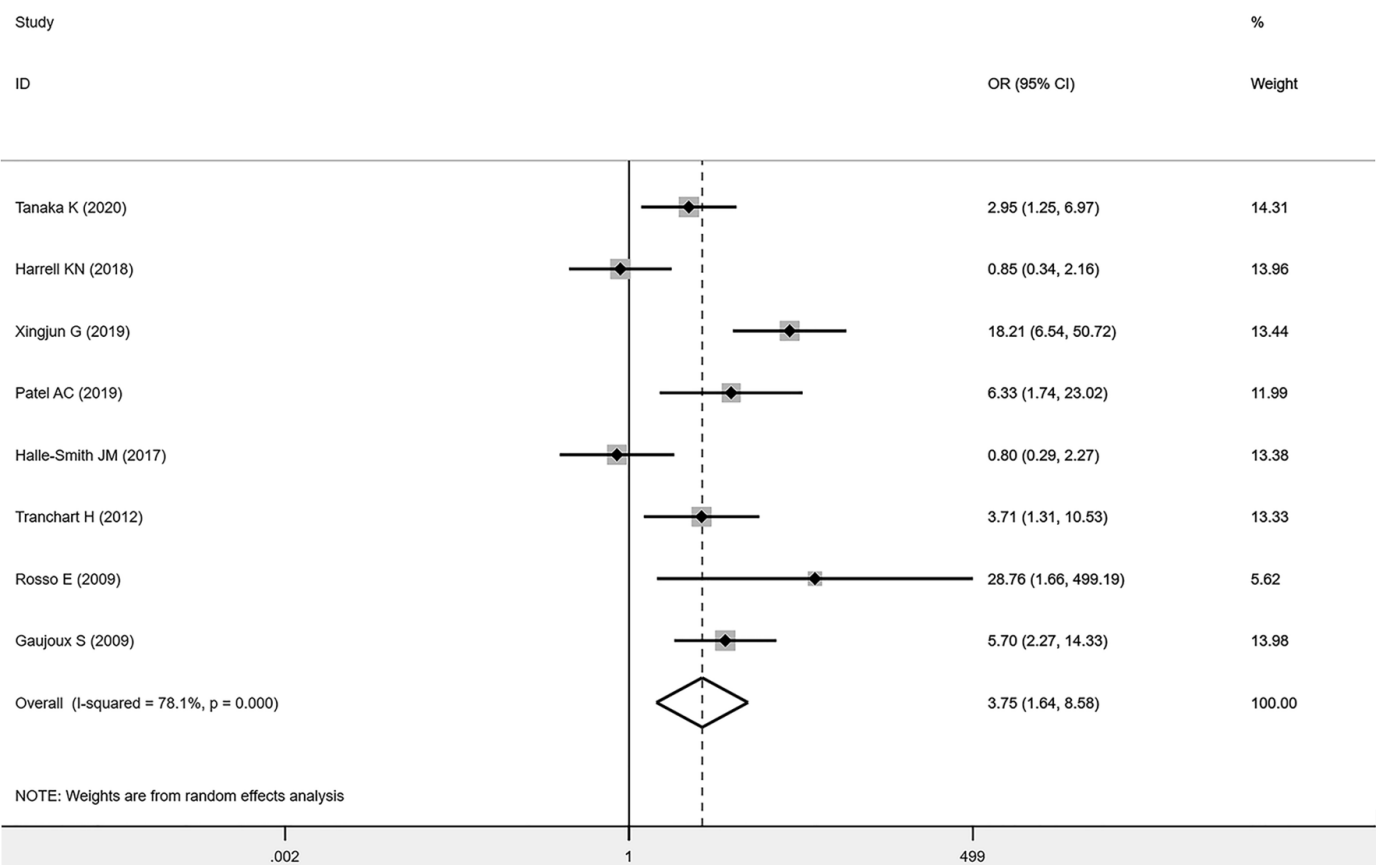
Forest plots of the effect of FP on POPF.

**Figure 5:**
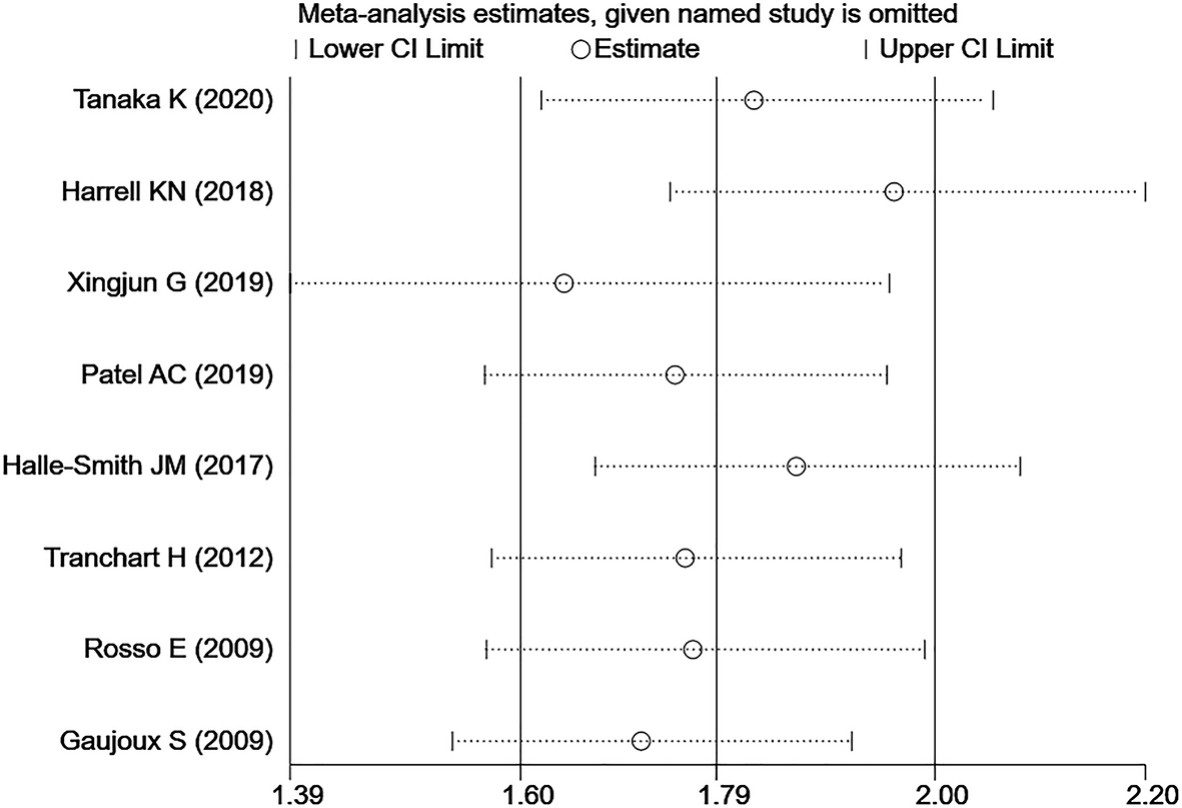
Sensitivity analysis of the association Association Between FP and POPF.

Eight studies (11–15, 17, 20, 21) described the number of patients with FP in detail, and a total of 1527 individuals were included in the analysis. A total of 228 patients suffered from POPF, and 157 of those patients had FP. Among the remaining 1299 non-POPF patients, 589 individuals were diagnosed with FP. FP was significantly associated with an increased incidence of POPF (OR=3.75; 95% CI: 1.64,8.58; P=0.002; I²=78) (**Figure 4**). Since the I² value >50%, which indicated that the heterogeneity was high, we conducted a sensitivity analysis (**Figure 5**) and a subgroup analysis (**Table 2**) to analyse the causes of heterogeneity. As shown in **Figure 5**, sensitivity analyses were performed on all included studies, and two studies showed large heterogeneity (12, 13). When all of the included studies were pooled, I² = 78% and P = 0.002. After one article (12) was removed, I² = 72.6% and P = 0.002. After the other article (13) was removed, I² = 67.4% and P = 0.005. After removing both articles, a sensitivity analysis was carried out with the remaining studies, and the results indicated an I²=57.8% and P=0. 037. Therefore, it was not difficult to determine that these two studies could significantly affect the heterogeneity of our results, and the reasons for the causes of heterogeneity need to be analysed.

**Table 2:** Subgroup analysis for the association association between FP and POPF.

Subgroups	No. of studies	Patients(n)	OR	95%CI	P value	I²(%)
**Male**	4 (11, 14, 15, 21)	403	1.72	1.06,2.78	0.03	51
**NAFPD diagnostic method**	8 (11–15, 17, 20, 21)	1527				
A	5 (11, 15–17, 21)	506	3.11	1.54,6.27	0.049	58
B	3 (12, 13, 20)	1021	6.61	0.51,86.4	<0.0001	91.2
**Regionalism**	8 (11–15, 17, 20, 21)	1527				
Asian	3 (11, 13, 14),	805	6.85	2.09,22.42	0.021	74
North America and Europe	5 (12, 15, 17, 20, 21)	722	2.49	0.89,6.93	0.003	74.9

**A**: These studies have a common pattern of diagnosis. The total score of pancreatic fatty infiltration was obtained by the addition of both perilobular and intralobular scores. Perilobular and intralobular fatty infiltration were scored as follows: 0: no fatty infiltration; 1: some adipocytes; and 2: numerous adipocytes. A total score of 0-2 was considered as a fat-free type, whereas a total score of 3-4 was regarded as a fatty infiltration type. **B:** Each study has different way of diagnosis.

Next, we performed subgroup analyses according to sex, age (which could not be successfully included due to the small number of studies and inconsistent statistical methods of data), FP diagnostic methods, and regional differences (**Table 2**). Among the included studies, four (11, 14, 15, 21) provided clear and extractable data on sex classification, and a total of 403 individuals were included in this study. There were 108 patients with POPF, of whom 74 were male, and the number of non-POPF patients was 295, of whom 172 were male. The results suggested that male sex was significantly associated with an increase in the incidence of POPF (OR=1.72; 95% CI: 1.06, 2.78; P=0.03; I²=51%). Eight studies were categorized into two groups according to the regions of the target countries: Asian (11, 13, 14) or European and North American (12, 15, 17, 20, 21). The results showed that FP was significantly associated with an increase in the incidence of POPF in Europe and North America (OR=2.49; 95% CI: 0.89,6.93; P=0.003; I²=74.9%) and Asia (OR=6.85; 95% CI: 2.09, 22.42; P=0.021; I²=74%), and the heterogeneity was not significantly different between the two groups. According to the different diagnostic methods of FP, we divided the eight studies into two groups: group A (11, 14, 15, 17, 21) and group B (12, 13, 20). Group A comprised all studies with the same diagnostic methods, and the inclusion criteria were as follows: The total score of pancreatic fat infiltration was obtained by adding the perilobular scores and the interlobular scores. Perilobular and intralobular fatty infiltration were scored as follows: 0: no fatty infiltration; 1: some adipocytes; and 2: numerous adipocytes. A total score of 0-2 was regarded as fat-free, whereas a total score of 3-4 was regarded as fatty infiltration. Group B, on the other hand, had different diagnostic modalities. P values were less than 0.05 in both group A (OR=3.11; 95% CI: 1.54, 6.27; P=0.049; I²=58%) and group B (OR=6.61; 95% CI: 0.51, 86.4; P<0.0001; I²=91.2%), suggesting that there was a significant correlation between FP and the occurrence of POPF. It should be noted that the 95% CI ranged on both sides of 1, and the heterogeneity was quite large in group B, which indicated that there was no statistical difference in group B, however, the P value was significant. This phenomenon may be related to the small sample size of group B and inconsistent classification standards, which eventually leads to the inconsistency of data. In addition, by combining the results of the sensitivity analysis and subgroup analysis, it could be concluded that the greater heterogeneity of this study may be due to differences in the diagnostic methods of FP in some studies. After excluding this part of the studies, the results were still reliable.

## Discussion

In 1931, Ogilvie (22) found for the first time that only 9% of the thin population had pancreatic fatty infiltration compared to 17% of the obese population at autopsy. Although a large number of researchers have focused their efforts on the study of nonalcoholic fatty liver disease (NAFLD) over the past few decades, FP did not become the focus of research until recently. Although the formation mechanism of FP is not yet clear, it has been shown that FP is reversible through weight loss or pharmacological treatment (23, 24).

To the best of our knowledge, PD is currently the main treatment for pancreatic cancer. Although surgical techniques and postoperative management have improved in recent years, with the mortality rate decreasing to approximately 5% in large-capacity hospitals compared to the previous period (25), the incidence of postoperative complications is still as high as 26.7% (26). However, the goal of clinicians is to find predictors of POPF so that high-risk patients can be identified before surgery to avoid the onset of POPF. Previous studies have shown that risk factors for POPF include a soft pancreas, small pancreatic duct, reduced regional blood supply, male sex, coexisting diseases, operation duration, type of pancreaticoenterostomy and surgical expertise (1, 4, 6, 27). Soft pancreatic parenchyma is the most widely accepted risk factor (28). Although the mechanism by which FP promotes the occurrence of POPF has not been elucidated, some studies have proposed a possible theory for its pathogenesis (29): First, during pancreatic and intestinal anastomosis reconstruction, a soft pancreas is more susceptible to ischaemia and injury than a hard pancreas. When suturing between the fragile pancreatic parenchyma and the plasma or muscle layer of the intestine or stomach, the pancreatic ducts and parenchyma are more susceptible to laceration and injury. Second, soft pancreas is often accompanied by small pancreatic ducts; as a result, it is usually not associated with pancreatic duct obstruction and rarely leads to ductal dilatation. Finally, and perhaps most importantly, the exocrine function of a soft pancreas is generally preserved, resulting in the increased secretion of pancreatic juice enriched with proteolytic enzymes. This can exacerbate tissue damage and impede healing of the incision over the long term. This lays the foundation for the occurrence of POPF. Although FP has not been described as the cause of POPF in previous studies, pancreatic lipid infiltration can significantly increase the softness of the pancreatic gland (30), which can lead to the occurrence of POPF.

In previous studies, the assessment of the softness or hardness of the pancreas was usually made by surgical palpation during the operation or histological evaluation of the specimen after the operation (31). Although ultrasound elastography (30) and fibre scanning (32) have recently been used to measure the elasticity of different tissues, to date, there is no unified measurement standard for the evaluation of pancreatic texture. Therefore, the purpose of our study was to identify an accurate and objective preoperative risk assessment index for POPF so that surgeons can customize appropriate management strategies for patients suffering from pancreatic cancer. In recent years, many researchers have found that “FP” is associated with a high risk of POPF (21, 31). Our results further confirm that FP can significantly increase the incidence of POPF. In addition, we conducted a subgroup analysis and found a significant correlation between FP and the increased incidence of POPF in all subgroups of FP. All of these findings suggest that FP may be an independent risk factor for POPF. At the same time, another result of our meta-analysis showed that the incidence of POPF was significantly increased in obese individuals (BMI >25 kg/m²), which is consistent with previous studies.

FP may be one of the most important risk factors for POPF that can be measured and controlled at an early stage. Since POPF is the main determinant of morbidity and mortality from severe complications after pancreatectomy, it is particularly important for identifying high-risk patients who are prone to POPF. Thus, for patients with FP or a BMI > 25 kg/m^2^, appropriate anastomosis type, external stents, and strict drainage management should be selected to reduce the risk of POPF.

Before surgery, how do we determine quickly and accurately whether a patient has FP? There are two main types of diagnostic methods available: histological examination and imaging examination. Because of the invasive nature of tissue biopsies and the disadvantages of a high rate of “false negatives”, if doctors want to determine whether the patient has FP before surgery, imaging is the best approach. To date, screening for FP is mostly performed *via* ultrasound. An ultrasound diagnosis of FP is generally obtained by comparing the echoes of the pancreas with those of other organs, such as the kidney, liver, or spleen. However, the pancreas and kidneys are not in the same acoustic window, and FP often occurs at the same time as fatty liver, which makes this diagnostic approach challenging. EUS compensates for the deficits of ultrasonography and has higher sensitivity and accuracy for the diagnosis of FP, so this kind of diagnostic method can be used for small-scale clinical screening.

Admittedly, although extensive, this study still has limitations. In our study, the heterogeneity in the meta-analysis of the incidence of POPF was high, which could be due to several reasons. First, the heterogeneity regarding rates across studies tended to be large, which is a problem in the vast majority of studies on rates. This is also a characteristic of meta-analyses of a single-group rate. Since only a single group of data is considered, the confidence intervals obtained for large-sample data are small, while the confidence intervals obtained for small-sample data are relatively large; this difference directly leads to significant differences in I^2^ and P values. Second, the degree of understanding of POPF differs among different regions, and the number of reports differs, resulting in large differences in survey results. Therefore, the investigation of the prevalence of POPF on a global scale still needs to be improved. Third, several papers included in this meta-analysis on the prevalence of POPF did not provide complete basic information; namely, some of them lacked the number of patients with FP, BMI, age, and so on. This limited our ability to conduct further meta-regression and subgroup analyses. All of these factors made it difficult to identify the sources of heterogeneity. However, our meta-analysis still has important clinical value. In addition, the pathological type of pancreatic cancer may have an impact on the incidence of POPF (33). However, fewer articles were included in the analysis because most articles did not describe the postoperative pathological classification in detail. The only three articles are not enough to support the needs of this meta-analysis (12, 17, 21), which leads to a lack of data on the potential high-risk and low-risk pathology of CR-POPF. It is also known that different types of surgery may affect the incidence of POPF. Unfortunately, among the 11 literatures included in the analysis, we found that only two literatures classified the types of surgery in detail (11, 16), and only PD surgery was performed in 7 literatures (12–15, 17, 20, 21), in addition, 2 literatures did not describe the type of surgery (18, 19). Therefore, the effect of surgical classification on POPF was not analyzed specifically. More clinical studies are needed to analyse the effect of pathological typing of pancreatic cancer on POPF in the future.

As a consequence, more extensive multicentre screening of POPF patients is needed in the future, as are prospective cohort studies, to further improve our understanding of this disease. In conclusion, our finding that FP is associated with the development of POPF will help clinicians better identify these high-risk patients.

In summary, FP and obesity can increase the incidence of POPF, and the early identification of high-risk patients can help to reduce postoperative complications.

## Data Availability Statement

The original contributions presented in the study are included in the article/supplementary material. Further inquiries can be directed to the corresponding author.

## Author Contributions

LZ: Writing – original draft. W-MX: Writing – original draft. C-PL: Writing – original draft. Y-WG: Writing – original draft. W-JG: Writing – review and editing. G-TL: Conceptualization and methodology. All authors contributed to the article and approved the submitted version.

## Funding

This study was supported by the National Natural Science Foundation of China (No. 82070668, 81801970), Major public health projects in Yangzhou: Screening projects of early gastrointestinal diseases (2018) and the National Natural Science Foundation of Yangzhou (No. YZ2018091).

## Conflict of Interest

The authors declare that the research was conducted in the absence of any commercial or financial relationships that could be construed as a potential conflict of interest.

## Publisher’s Note

All claims expressed in this article are solely those of the authors and do not necessarily represent those of their affiliated organizations, or those of the publisher, the editors and the reviewers. Any product that may be evaluated in this article, or claim that may be made by its manufacturer, is not guaranteed or endorsed by the publisher.
